# The Mitochondrial Unfolded Protein Response: A Novel Protective Pathway Targeting Cardiomyocytes

**DOI:** 10.1155/2022/6430342

**Published:** 2022-09-21

**Authors:** Jinfeng Liu, Xinyong He, Sicheng Zheng, Aisong Zhu, Junyan Wang

**Affiliations:** ^1^Guang'anmen Hospital, Chinese Academy of Traditional Chinese Medicine, Beijing 100053, China; ^2^Liaoning University of Traditional Chinese Medicine, Shenyang 110032, China; ^3^School of Basic Medical Sciences, Zhejiang Chinese Medical University, Hangzhou 310053, China; ^4^School of Pharmaceutical Sciences, Guangzhou University of Chinese Medicine, Guangzhou 510405, China

## Abstract

Mitochondrial protein homeostasis in cardiomyocyte injury determines not only the normal operation of mitochondrial function but also the fate of mitochondria in cardiomyocytes. Studies of mitochondrial protein homeostasis have become an integral part of cardiovascular disease research. Modulation of the mitochondrial unfolded protein response (UPRmt), a protective factor for cardiomyocyte mitochondria, may in the future become an important treatment strategy for myocardial protection in cardiovascular disease. However, because of insufficient understanding of the UPRmt and inadequate elucidation of relevant mechanisms, few therapeutic drugs targeting the UPRmt have been developed. The UPRmt maintains a series of chaperone proteins and proteases and is activated when misfolded proteins accumulate in the mitochondria. Mitochondrial injury leads to metabolic dysfunction in cardiomyocytes. This paper reviews the relationship of the UPRmt and mitochondrial quality monitoring with cardiomyocyte protection. This review mainly introduces the regulatory mechanisms of the UPRmt elucidated in recent years and the relationship between the UPRmt and mitophagy, mitochondrial fusion/fission, mitochondrial biosynthesis, and mitochondrial energy metabolism homeostasis in order to generate new ideas for the study of the mitochondrial protein homeostasis mechanisms as well as to provide a reference for the targeted drug treatment of imbalances in mitochondrial protein homeostasis following cardiomyocyte injury.

## 1. Introduction

Cardiomyocyte injury and activity decline are determined by multiple factors and contribute to the pathological mechanisms of various cardiovascular diseases [1]. This is the primary cause of occurrence and development of cardiovascular diseases and death in humans [2, 3]. Targeted protection and activity regulation of cardiomyocytes are important clinical problems. Because mitochondria provide more than 90% of the adenosine triphosphate (ATP) required by the heart to maintain homeostasis and systolic function; the myocardium is particularly vulnerable to mitochondrial energy metabolism dysfunction [4].

Mitochondria are not only important for pathological mechanisms involved in myocardial ischemia-reperfusion injury, but also a potential drug target for myocardial protective intervention after reperfusion injury [5]. Consequently, the steady state and correct folding of mitochondrial proteins are issues of primary importance in current research. When mitochondria undergo ischemia or reperfusion stress injury, proteins in the mitochondria misfold and the mitochondrial unfolded protein response (UPRmt) is activated [6]. The UPRmt mediates the regulation and folding of the mitochondrial proteome and mitochondrial quality control [7]; it is a classic retrograde signal transcription response. Protein homeostasis is important in the physiological and pathological processes of various human diseases [8]. Protein homeostasis is regulated by mitochondrial protein homeostasis, which is mainly composed of mitochondrial protein [9]. The balance between the quality and quantity of mitochondrial proteins mainly depends on the presence of specific mitochondrial heat shock proteins (HSP60 and HSP10) and proteases encoded by nuclear DNA (ATP-dependent CLP proteolytic subunit 1 CLPP, Lon protease) in the mitochondria [10, 11]. The correct folding and steady-state mechanisms of these proteins are related to mitophagy/mitochondrial fission and mitochondrial biosynthesis, which are required for maintenance of the stability of mitochondrial quality and quantity [12]. Once the external environment or stress stimulation (ischemia, hypoxia, high glucose, or inflammation) changes, it usually causes abnormalities in the protein structure or signal transmission function in the mitochondria [13]. If the mitochondrial structure is destroyed or misfolded proteins cannot be degraded in time and accumulate in large quantities, the UPRmt is induced. This leads to the activation of apoptosis or the necrotic apoptosis signal pathway, which, in turn, can lead to cellular activity decline or cell death [6]. The UPRmt can be induced in many cardiovascular diseases, such as heart failure, septic cardiomyopathy, and acute myocardial ischemia [14], making it a potentially valuable target for therapeutic intervention with cardioprotective drugs.

## 2. The UPRmt in Intracellular Environmental Homeostasis of Cardiomyocytes

Mitochondria are the central hub of signal transmission in various cellular energy metabolic, physiological, and pathological processes [15]. They continuously fuse and divide in the course of important processes such as energy supply, cellular aerobic respiration/anaerobic glycolysis, mitophagy and self-digestion by lysosomes, and programmed cell death, in order to maintain the normal function and state of cells [16]. Mitochondrial function depends mainly on the organelle proteome. Approximately 99% of mitochondrial proteins are synthesized as precursors by cytoplasmic ribosomes and are introduced into target organelles through protein transposase [17]. Mitochondria also retain their own genome. ATP synthase and respiratory chain complexes I, III, and IV are proteins with dual genetic sources [18]. The proteins encoded by mitochondria constitute the response center and are associated with many nuclear-encoded proteins [19]. The coordinated expression of nuclear and mitochondrial genes and coordinated assembly of protein complexes together form the steady-state mechanism of mitochondrial proteins [20]. The quality and quantity of mitochondria directly determine the normal maintenance of mitochondrial function (including energy metabolism, ATP production, and mitochondrial redox reaction), which directly affects the function and state of cardiomyocytes [21]. Cells will evolve methods to monitor mitochondrial function and quickly respond to mitochondrial oxidative stress, calcium overload stress, and other stresses to restore organelle function [22]. This type of pathway is usually called a reverse response or reverse signal transduction pathway because the upstream signal of this process starts in the mitochondria and is transmitted to the cytoplasm and nucleus, protects mitochondrial and cellular gene transcription, and directly or indirectly affects mitochondrial protein homeostasis [23]. In the process of mitochondrial dysfunction due to unfolded/misfolded proteins in the mitochondria, cells use a transcription mechanism called the UPRmt to maintain homeostasis of the intracellular environment and repair the mitochondria whose quantity or quality is affected by stress [24]. This response is an important component of the reverse signal transduction pathway in cells.

The precise regulation of the UPRmt in cells is related to the survival state of cells [25]. The UPRmt can induce ATFs-1 to promote oxidative phosphorylation under mitochondrial stress [26]. UPRmt protease (yme1l/CLPP) can promote the recovery of respiratory chain function and help maintain oxidative phosphorylation in stressed mitochondria through the action of ATFs-1 [27]. The UPRmt is finely regulated by multiple signal transduction pathways and can complete communication between the nucleus and mitochondria in different organisms [28]. The activation and regulation mechanisms of the UPRmt differ among diseases or pathological states. During mild mitochondrial dysfunction caused by appropriate stress regulation, UPRmt activation can promote development and prolongation of life, which indicates that enhancing UPRmt activation could be an effective treatment [29]. Interestingly, in cellular stress and cardiac disease states (such as myocardial ischemia/hypoxia injury), prolonged UPRmt activity may lead to mitochondrial DNA damage and biosynthetic dysfunction [30]. In the injury process of various cell stress states, limiting or depriving the overactivation of UPRmt may be one of the most effective ways to improve mitochondrial function. The dual regulation of the UPRmt may also be closely related to the two-way regulation of mitophagy.

In addition to the above-mentioned mechanisms, reactive oxygen species- (ROS-) mediated oxidative stress injury is another main cause of dysregulated homeostasis and UPR dysfunction in cardiomyocytes. Existing forms of ROS usually include superoxide anions (O_2_-), hydroxyl radicals (OH·), peroxide free radicals (ROO·), and hydrogen peroxide (H_2_O_2_) [29]. In cardiomyocytes, the mitochondria may be the main source of ROS production. In mitochondria, the electron transport chain (ETC) is the main mode of ROS generation, caused by nonspecific electron leakage, and is driven by the activity of ETC complexes (mainly complexes I and III). In addition, the mitochondrial succinate dehydrogenase (SDH) complex is capable of producing superoxide in the absence of electron acceptors. ROS production is balanced by ROS scavenging by the antioxidant system within mitochondria. It can be reduced to H_2_O by catalase or an antioxidant system consisting of glutathione, glutathione peroxidase (GPX), peroxidase (PRX), and thioredoxin (TRX) [20]. The mechanism is determined by the redox state and availability of reducing equivalents within the mitochondria. Excessive mitochondrial ROS leads to mitochondrial dysfunction in cardiomyocytes, affects ATP production, leads to dysregulation of mitochondrial energy metabolism, induces cell death, and ultimately leads to myocardial contractile dysfunction [26]. The regulatory mechanism for ROS-mediated injury may be related to the UPRmt.

Mitochondria are vital for fundamental eukaryotic processes. The mitochondria are relatively complex and dynamic in cardiomyocytes. They have many unique properties that are associated with cell signaling and programmed cell death. These properties enable the mitochondrial redox balance response system to respond more flexibly to various stimuli and changing environments, making ROS generation and accumulation relatively stable. This process mainly involves maintaining proper mitochondrial oxidative balance and metabolic function during the physiological phase and stress stimulation period of the cell [28]. ROS-mediated mitochondrial oxidative stress disrupts mitochondrial proteostasis and mediates the dysfunction of mitochondrial unfolded protein responses. Highly conserved mitochondrial proteases and molecular chaperones prevent the formation of toxic protein aggregates and degrade damaged proteins by enabling proper protein folding and assembly. When the protease chaperone machinery is overwhelmed, the UPRmt transcriptional response is initiated [28]. Many studies have found that excess ROS can impair the regulatory mechanisms of the UPRmt. This further affects mitochondrial quality control, resulting in a decrease in the level of mitophagy and a breakdown in the balance between mitochondrial fusion and fission. Continuous fusion and fission cycles lead to mitochondrial membrane dysfunction and morphological structural changes in intact mitochondria and activate caspase-related apoptosis. Therefore, ROS-induced mitochondrial oxidative stress damage can directly or indirectly lead to dysregulated mitochondrial quality control and UPRmt dysfunction, leading to myocardial cell damage.

## 3. Molecular Regulatory Mechanisms of the UPRmt in Cardiomyocyte Physiology

Mitochondria contain more than 1000 different types and functions of proteins. Mitochondrial proteins are predominantly encoded by nuclear DNA and synthesized on cytoplasmic ribosomes for delivery to the outer mitochondrial membrane (OMM) or are imported via translocases to the inner or outer mitochondrial membranes. Homeostasis between mitochondria and the synthesis, transport, localization, expression, and degradation of proteins is critical for mitochondrial structure and function and is also an important factor that governs the homeostasis of cardiomyocytes and interactions between organelles. To ensure the coordinated operation of this system with a wide variety of proteins, mitochondria have a special protein quality control system to supervise protein translation. This system mediates protein folding, assembly, and import and export [28].

The mitochondrial function of cardiomyocytes depends mainly on the organelle proteome. Approximately, 99% of mitochondrial proteins are synthesized as precursors of cytoplasmic ribosomes and are imported into target organelles through protein translocases. Mitochondria also retain their own genome and are a part of the respiratory chain and ATP synthase, where misfolded proteins accumulate. Cardiomyocytes can use impaired protein inputs as sensors of mitochondrial dysfunction and activate stress responses. Protein misfolding within the matrix is sensed as a robust mitochondrial stress adaptation process [10]. Stress responses to unfolded or misfolded mitochondrial proteins induce transcriptional programs. Then, by altering gene expression to increase protein folding capacity in the mitochondria, mitochondrial protein homeostasis and mitochondrial function are maintained or enhanced. Therefore, the mitochondrial unfolded protein response is one of the adaptive pathways of cardiomyocytes activated by stress, such as inflammation, high glucose, high lipid, and hypoxia/ischemia.

**Figure 1:**
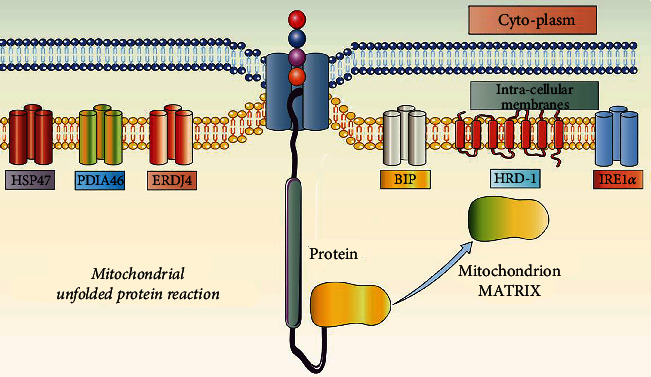
Regulatory mechanism of mitochondrial unfolded protein response. Mitochondrial unfolded protein response plays an important regulatory role in cell physiological activities. Under stress stimulation conditions such as ischemia/hypoxia or inflammation, the homeostasis of intracellular environment and mitochondrial homeostasis will be further destroyed. Oxidative phosphorylation (OXPHOS) disturbance, excessive ROS, impaired complex assembly (mitotic nucleoprotein imbalance), and accumulation of misfolded proteins further activate HSP47/PDIA46/ERDJ4 under the induction of mitochondrial proteins. Meanwhile, BIP/HRD-1 and IRE1*α* will activate in the intima. Together with HSP47/PDIA46/ERDJ4, it regulates the homeostasis of mitochondrial matrix and mitochondrial proteins, which is an important mechanism affecting mitochondrial protein homeostasis.

As shown in Figure 1, the UPRmt plays diverse and complex roles in the regulation of molecular mechanisms. In the early stages, UPRmt only plays a role in a single damaged mitochondrion and can be activated without cell stress [26]. The UPRmt can affect mitochondrial protein folding by regulating the input and translation of mitochondrial proteins [31]. Human high mobility group (HMG) box transcription factor 1 can transfer to the mitochondria and bind to mitochondrial DNA, play the same function as mitochondrial transcription factor A (TFAM) encoded by nuclear genes, maintain the steady-state mechanism of mitochondrial DNA, and ensure protein stability [32]. In addition, when the UPRmt is induced by HSP90 and LONP1 inhibitors, the transcript and protein levels of mitochondrial ribonuclease P protein 3 decrease rapidly. The transcription level of mitochondrial precursor RNA also decreases significantly, and this can eventually lead to the reversible reduction of local mitochondrial translation [25].

The UPRmt signaling pathway, involved in the regulation of mitochondrial proteins, is chiefly related to CCAAT enhancer binding protein (C/EBP) homologous protein (CHOP) and activating transcription factors (ATFs) 4 and 5. When ornithine transcarbamylase is mutated, the mitochondrial matrix, heat shock proteins HSP60 and HSP10, human mitochondrial DNA haplogroup J (mtDNAj), and mitochondrial protease CLPP are overexpressed, whereas the molecular chaperones of the cytoplasm and endoplasmic reticulum are not activated, and the increased expression of these proteins in the mitochondria depends on the transcription factors CHOP and C/EBP [33]. Bioinformatic analysis revealed the presence of CHOP and C/EBP binding domains on the promoters for HSP60, HSP10, mtDNAj, and CLPP. As CHOP and C/EBP can induce the endoplasmic reticulum unfolded protein response, Shen et al. [34] speculated that the specificity of the mitochondrial stress response may be caused by the selective induction of CHOP by activator protein 1.

ATF4 is a transcription factor that contains a basic leucine zipper. It can shuttle from the mitochondria to the nucleus during regulation of mitochondrial homeostasis. ATF4 is a key regulator of the induction of UPRmt and maintains homeostasis of mitochondrial proteins [35]. Recently, researchers found that ATF4 knockout led to a decrease in the expression of *LONP1*, *HSP10*, and *HSP60* in alveolar epithelial cells. ATF5, a gene homologous to ATFs-1, also plays a role in regulating the mitochondrial stress response in cells. Similar to ATFs-1, ATF5 is also present in the mitochondria and nucleus [36]. ATF5 induces not only the UPRmt of nematodes but also HSP60 and HSP70, promotes the proliferation of cells under stress, and assists in the recovery of mitochondrial function [37].

UPRmt activation is also related to the sirtuin 3 (SIRT3)-forkhead-like transcription factor o3a (FOXO3a)-superoxide dismutase 2 (SOD2) pathway. NAD-dependent deacetylase 3 (SIRT3) is the main deacetylase that promotes aerobic metabolism in the mitochondrial matrix and regulates the production of mitochondrial ROS [38]. In the mitochondrial matrix, upregulation of SIRT3, SOD2, and peroxidase can promote the conversion of superoxide to water. Antioxidant activity is directly related to protein homeostasis. The reduction in superoxide levels limits the oxidation and misfolding of proteins in the mitochondria. Some researchers found that after mitochondrial protein toxic matrix stress, the expression of SIRT3 was upregulated, resulting in the transport of FOXO3a from the cytoplasm to the nucleus and the induction of its transcription targets SOD2 and catalase, causing an antioxidant response and reducing ROS production; SIRT3 can coordinate two pathways, mediating antioxidant mechanisms and mitochondrial autophagy. Inhibition of SIRT3 in cells undergoing proteotoxic stress can seriously damage the mitochondrial network and lead to cell death [39]. These results indicate that SIRT3 distinguishes organelles under moderate pressure from those that are irreversibly damaged. SIRT3 acts as a tumor suppressor during transformation. The results revealed the dual role of SIRT3; it can participate in the regulation of the UPRmt through mitochondrial autophagy, but its regulatory function is different.

Estrogen receptor *α* (ER*α*), an important transcription factor in cells, is also one of the regulatory pathways mediating UPRmt [40]. In the mitochondrial intermembrane space (IMS), the expression of mutant endonuclease G activates another UPRmt signaling pathway, resulting in the accumulation of misfolded endonuclease G in IMS, which is independent of the matrix UPRmt and does not induce CHOP or HSP60 [41]. IMS stress also leads to an increase in ROS, which leads to the phosphorylation of ROS-dependent protein kinase B (PKB or Akt), which in turn leads to ER*α* activation [42]. ER*α* activation induces nuclear respiratory factor 1 (Nrf1) expression. It can be suggested that IMS can induce ER stress and stimulate the cell-protective response to maintain mitochondrial integrity [43]. The UPRmt, which includes CHOP, SIRT3, and ER*α* activity, can counteract disturbances in mitochondrial protein homeostasis due to oxidative stress, eliminate misfolded protein stress, and maintain mitochondrial protein homeostasis [20]. Currently, the key regulatory factors of the UPRmt are still controversial, and relationships among CHOP, ATF4, and ATF5 are also under investigation.

## 4. Molecular Biological Mechanisms of the UPRmt

### 4.1. Hsp10/Hp60/HSP70 and HSF1 Pathways

Classical proteases that induce mitochondrial protein folding or protein chaperone formation can prevent mitochondrial protein folding [44]. Chaperones are mainly composed of HSP10, HSP60, mitochondrial HSP70 (mtHSP70), and other molecular chaperones [45]. Chaperone HSP60 plays an important regulatory role in mitochondrial protein homeostasis by mediating protein folding [46]. The transfer of protein precursors in the cytoplasm to the mitochondrial matrix requires participation of the molecular chaperone mtHSP70 [47]. mtHSP70 directly interacts with the mitochondrial inner membrane translocation enzyme 23 complex to insert the protein into the mitochondrial matrix and prevent reverse diffusion of the protein into the cytoplasm. Concurrently, mtHSP70 can assist in the correct processing of proteins in the channel and matrix [47]. The mitochondrial subtypes of the HSP90 and HSP10 complexes are also involved in the folding of matrix-localized peptides [48]. Under the action of chaperonin 10 (cpn10), the protein is induced to obtain a suitable conformation [49].

Studies have shown that HSP10 is found in the coronary effluent of an IPC-treated heart, which has the potential for cardiac protection [50]. An isolated mitochondrial model was used to characterize the effect of exogenous HSP10 on myocardial mitochondrial function after ischemia-reperfusion injury. Incubation with HSP10 under hypoxia/reoxygenation conditions in freshly isolated mitochondria prevented the hypoxia/reoxygenation-induced production of mitochondrial ROS. Mitochondria are targets of HSP10-induced cardioprotection. HSP10 can directly act on mitochondria, improve hypoxia/reoxygenation injury, and protect cardiomyocyte mitochondria through ATP activation.

The heat shock response is mainly coordinated by heat shock transcription factor 1 (HSF1). During the regulation of cell physiology and pathology, HSF1 can increase the folding, decomposition, and degradation ability of proteins by improving the expression level of molecular chaperones [51]. HSF1 is essential for induction of mitochondrial chaperones during the UPRmt. The heat shock response can trigger single-stranded DNA binding protein 1 (SSBP1), participate in the replication of mitochondrial DNA, and directly interact with HSF1. HSF1 can recruit SSBP1 to promoters encoding cytoplasmic/nuclear and mitochondrial chaperone genes to maintain cellular protein homeostasis [52]. In addition, the heat shock response can further affect the preferential binding state of HSP16 and HSP70 genes, stimulate different degrees of the UPRmt, and protect mitochondrial function.

### 4.2. ER*α*-Nrf1-HTRA2 Pathway

The UPRmt mediated by the ER*α*-NRF1-HTRA2 pathway is mainly directed toward the production of the inner mitochondrial IMS and is a cytoprotective response that helps maintain mitochondrial integrity [53]. Mitochondrial IMS is an important hub for mitochondrial energy metabolism and protein transport, and more than 100 proteins, coordinately localized in this sub-compartment, mediate important mitochondrial functions [54]. Mitochondrial IMS has no heat shock protein and has fewer proteases and antioxidant enzymes than are present in the matrix. ROS are generated in large quantities in the mitochondrial IMS; hence, the IMS is a very sensitive sensor for misfolded proteins [55].

When homeostasis in the mitochondrial IMS is disturbed or a stress response occurs, excessive accumulation of ROS and phosphorylation of protein kinase B (AKT) can activate the ER*α* pathway and induce expression of ER*α* [56]. This activation can further upregulate the transcription of the mitochondrial regulator Nrf1 and the mitochondrial inner membrane space protease HTRA2, upregulate proteasome activity, and promote degradation of misfolded proteins [36].

In the regulation of mitochondrial protein homeostasis, an excessive folding response can lead to excessive production of ROS [57]. However, excessive ROS can lead to mitochondrial oxidative stress and mitochondrial electron transport chain dysfunction; thus, mitochondrial IMS is a relatively sensitive region of the UPRmt, and targets in mitochondrial IMS may be at a higher risk of misfolding than proteins in other subcellular compartments.

### 4.3. ATF4/ATF5-CHOP Pathway

The UPRmt is mainly localized in the mitochondrial matrix and is regulated by ATF4, ATF5, and CHOP, which are homologous to the *Caenorhabditis elegans* transcription factor ATFS-1 [58]. CHOP is part of an integrated stress response that activates the transcription of mitochondrial chaperones and proteases. Many mitochondrial stressors unrelated to misfolded proteins inhibit mitochondrial membrane potential translation and rapidly induce CHOP responses without chaperone activation [59]. CHOP- and ATF4-induced ATF5 is involved in reverse signaling of the UPRmt to the nucleus. ATF5 is localized to the mitochondria and translocates to the nucleus during mitochondrial stress, where it transcriptionally upregulates mitochondrial chaperones and proteases [60]. The integrated stress response during stress leads to phosphorylation of eukaryotic initiation factor 2*α* (eIF2*α*), a process that promotes ATF4, ATF5, and CHOP activity [61].

Cells respond to stress by transcriptionally activating genes [62, 63]. Although ATF4 and CHOP may regulate cell survival through the UPRmt, there is currently a lack of understanding of coregulatory factors due to the nonspecific nature of CHOP itself. Neither ATF4 nor CHOP is sufficient to elicit a typical UPRmt transcriptional response, which may be dependent on additional signaling factors [64]. Induction of CHOP cannot simply be used as an indicator for the induction of the UPRmt, and CHOP is also closely related to ER stress and ER autophagy-mediated cell death. Therefore, the relationship between CHOP and ATF4 transcription factors during mitochondrial stress and the pathways specifically activated by mitochondrial perturbation have not been fully elucidated.

### 4.4. Sirt3-FOXO3a-SOD2 Pathway

The Sirt3-FOXO3 manganese SOD2 pathway mainly plays an antioxidant role and pivotal role in the UPRmt [65]. In mammals, the sirtuin family is implicated in the regulation of metabolic diseases, cardiomyocytes, and vascular endothelial cell growth and development [66]. Sirt3 is mainly localized in the mitochondrial matrix and participates in mitochondrial energy metabolism by regulating energy metabolism substrates [67]. It also regulates mitochondrial redox balance and protects cells from ROS-mediated oxidative stress. When mitochondria show a certain level of dysfunction under stress, misfolded proteins gradually accumulate [68]. At this time, the Sirt3-FOXO3a pathway is activated, and Sirt3 can exert mitochondrial antioxidant effects by controlling the activity and localization of the transcription factor FOXO3a [39]. Sirt3 levels increase in response to toxic stress from protein unfolding in the mitochondrial matrix, and FOXO3a is dependent on Sirt3 deacetylation to promote its nuclear translocation and transcriptional activity [69]. When ROS-mediated oxidative stress is severe, FOXO3a can also target the mitochondria and nucleus to regulate the mitochondrial antioxidant system dominated by SOD2 and catalase, and inhibit the occurrence and development of mitochondrial oxidative stress [70].

Therefore, the activities of Sirt3, SOD2, and FOXO3a are directly related to mitochondrial protein homeostasis, and the synergistic effect of Sirt3 and SOD2/FOXO3a in the mitochondria limits the oxidation and misfolding of proteins in the mitochondria [71]. Because of the complex regulatory mechanism of Sirt3 and many targeted regulatory pathways, a study has proposed that Sirt3 activation may be closely related to ROS-induced mitochondrial oxidative stress damage, but not directly related to mitochondrial folded protein responses [53]. The mechanisms by which the Sirt3/FOXO3a/SOD2, ATF4/ATF5-CHOP, and ER*α*-NRF1-HTRA2 pathways regulate the mitochondrial unfolded protein responses are different. Sirt3 has regulatory functions in the UPRmt, mitochondrial oxidative stress, and mitophagy. The specific molecular targets related to Sirt3 remain to be further studied and explored.

## 5. Protective Effect of the UPRmt on Pathological Damage of Cardiomyocytes

**Figure 2:**
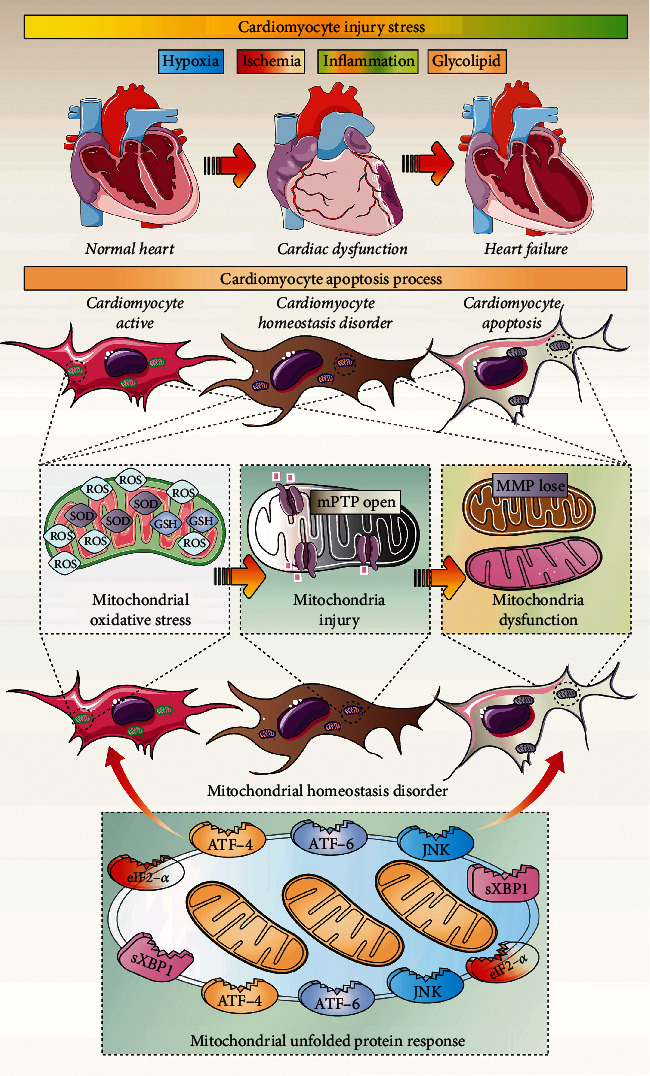
UPRmt is involved in cardiomyocyte mitochondrial homeostasis dysregulation and cardiomyocyte apoptosis. Mitochondrial ROS-induced mitochondrial oxidative stress is activated during the initial stages of myocardial injury and cardiomyocyte apoptosis. The mitochondrial membrane permeability transition pore (mPTP) is further activated, which induces an imbalance between the inner and outer mitochondrial environment, which in turn leads to a massive loss of mitochondrial membrane potential (MMP) and dysfunction of mitochondrial energy metabolism. This process activates the mitochondrial pathway of apoptosis, which in turn leads to cell death or apoptosis/necroptosis. UPRmt can be further activated under mild stress stimulation. Repairing mildly compromised mitochondrial oxidative stress damage or dysfunction, UPRmt can clear misfolded proteins (chaperones) or cleavage proteases. Unrepairable mitochondria may further undergo mitophagy, and mitochondrial DNA renewal and regeneration occurs through mitochondrial dynamics and mitochondrial biosynthesis.

UPRmt exerts a protective effect against myocardial cell injury caused by various cardiovascular diseases and stress states. The protective effects of the UPRmt on cardiomyocytes under stress include increasing ATP production, inhibiting oxidative stress injury induced by excessive accumulation of ROS, inhibiting the release of apoptotic factors, inhibiting calcium overload, and inhibiting the abnormal opening of the mitochondrial membrane permeability transition pores [72]. As shown in Figure 2, moderate UPRmt activation clears damaged mitochondrial proteins and maintains mitochondrial homeostasis and function [73]. The beneficial effects of the UPRmt on cells are also reflected in the maintenance of mitochondrial energy metabolism, maintenance of mitochondrial electron transport chain function, and prevention of the release and activation of proapoptotic factors [74]. The UPRmt can interact with mitochondrial quality control in the mitochondrial homeostasis mechanism of cardiomyocytes and can lead to changes in mitochondrial quality and quantity, thereby mediating the dysregulation of mitochondrial homeostasis [75]. Mitochondria produce most of the cellular energy and are targeted by various pathogens during infection [26].

The UPRmt, an adaptive transcriptional program regulated by ATFS-1, induces the expression of genes that promote mitochondrial recovery [76]. The mitochondrial heat shock proteins HSP60 and HSP10, which mediate the activation and regulation of UPRmt, form mitochondrial chaperone protein complexes that improve cardiomyocyte activity under ischemic conditions. Overexpression of HSP60 or HSP10 can increase ATP production and the activity of complexes III and IV in the mitochondria. Inhibiting mitochondrial cytochrome C release while reducing the activity of caspase 3 protects cardiomyocytes from apoptosis, suggesting that the antiapoptotic protective mechanism of cardiomyocytes is related to the UPRmt mediated by HSP60 and HSP10 [77].

Studies have found that oligomycin- or doxycycline-induced UPRmt can reduce myocardial infarct size in wild-type (WT) mice, and ATF5-mediated UPRmt can also provide protection against acute ischemia-reperfusion injury [78]. Regarding the regulation of the cardiomyocyte hypertrophic stress response, the UPRmt can effectively counteract pathological cardiac hypertrophy. Mice were subjected to transverse aortic coarctation (TAC) surgery [30]. siRNAs targeting PGC-1*α* and ATF5 were used to determine the mechanism of action of the UPRmt. Tetrahydrocurcumin supplementation upregulated the UPRmt effector mechanism, inhibited TAC-induced oxidative stress injury, improved cardiac contractile function, and inhibited cardiomyocyte hypertrophy and myocardial fibrosis compared with TAC-operated WT mice. Knockdown of PGC-1*α* can inhibit UPRmt activation and the cardioprotective effect of tetrahydrocurcumin. The mechanism of the protein interaction between PGC-1*α* and ATF5 is also indicated. Experimental results suggest that PGC-1 and ATF5 can partially activate UPRmt, thereby mediating the cardioprotective effect.

Experiments with SIRT3 have also found that SIRT3 ablation is associated with cardiovascular diseases [79]. SIRT3 ablation causes coronary microvascular dysfunction after myocardial ischemia and impairs cardiac recovery. SIRT3^-KO^ mice developed significant coronary microvascular dysfunction with reduced coronary flow reserve. Compared with WT mice, SIRT3^-KO^ mice had a more severe reduction in cardiac ejection fraction and significantly decreased cardiomyocyte, pericyte, and vascular endothelial cell activities. Interestingly, SIRT3 overexpression restored cardiac function in mice after myocardial infarction. The above findings reveal the underlying mechanism of SIRT3 in cardiac insufficiency and recovery of myocardial tissue or cardiac ejection function after myocardial infarction [79]. Related studies on quercetin have found that improvement of mitochondrial energy metabolism, mitochondrial fusion/fission balance, and mitochondrial biosynthesis activation is involved in the protective mechanism of cardiomyocytes. Through regulation of the above mechanisms, it can inhibit the inflammatory response and oxidative stress accompanied by myocardial fibrosis and cardiac function injury at the same time.

Furthermore, TAC-induced cardiac injury can inhibit the activation of SIRT5 and increase IDH2 succinylation at the mitochondrial level. Quercetin promotes the desuccinylation of IDH2 by increasing SIRT5 expression. SIRT5 and SIRT3, as SIRT family molecules, can both have protective effects on cardiomyocytes or coronary microvascular endothelial cells, and both can regulate mitochondrial homeostasis, but their regulatory mechanisms in the UPRmt response need further verification [80].

## 6. Bidirectional Regulation of UPRmt: A Double-Edged Sword

**Figure 3:**
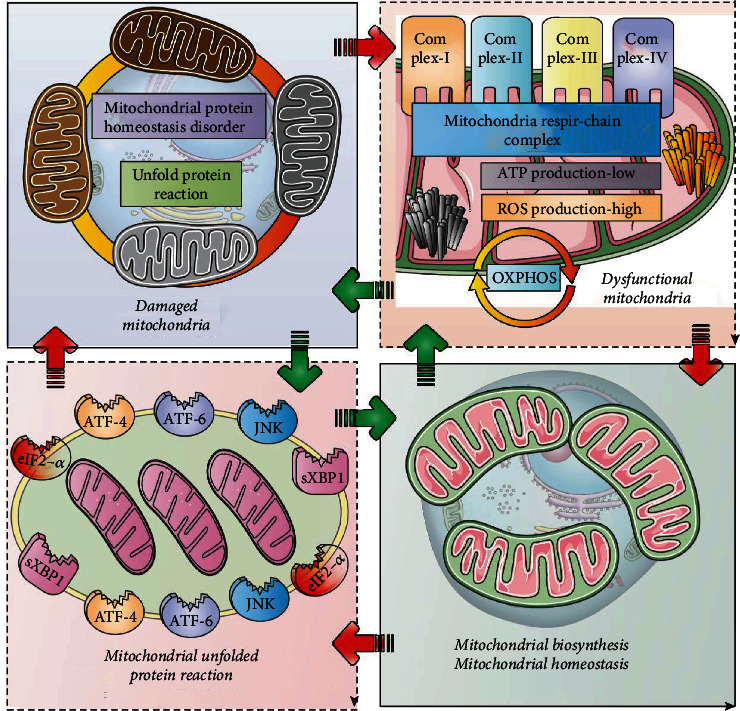
Bidirectional regulation of UPRmt in cardiomyocyte mitochondrial injury and mitochondrial biogenesis, a double-edged sword. With mitochondrial aging and stress-induced mitochondrial death, the function of the mitochondrial respiratory chain will be severely affected, which will lead to mitochondrial ATP synthesis dysfunction, excessive accumulation of mitochondrial ROS, and then mitochondrial respiratory complexes dysfunction. It will eventually lead to mitochondrial oxidative phosphorylation dysfunction and mitochondrial tricarboxylic acid cycle dysfunction, which may be an important cause of mitochondrial protein dysfunction and mitochondrial damage. In the state of mild mitochondrial damage, UPRmt is activated, which in turn affects mitochondrial biosynthesis and mitochondrial homeostasis, providing a guarantee for the balance of intracellular homeostasis. In states of mild mitochondrial damage or dysfunction, UPR is beneficial as it reduces reactive oxygen species (ROS) production and increases ATP activity to protect mitochondrial function.

As shown in Figure 3, the UPRmt has a protective effect against cardiomyocyte injury caused by various cardiovascular diseases and stress states [81]. However, a series of relatively contradictory studies have also found that blocking the UPRmt pathway can improve cardiomyocyte injury under various stress conditions to varying degrees. Using only gene overexpression/knockout models, differentiating the cardiotoxic and cardioprotective effects of UPRmt is difficult [82]. Therefore, it is inferred that UPRmt can play a bidirectional regulatory role in stress-induced mitochondrial damage and mitochondrial biogenesis.

Related studies on the UPRmt and myocardial ischemia-reperfusion injury found that UPRmt activation could alleviate myocardial ischemia-reperfusion injury under the action of oligomycin [83]. Supplementation with the UPRmt inducer oligomycin effectively reduced myocardial infarction and improved systolic/diastolic function. Ischemia-reperfusion injury causes a rapid increase in biomarkers of myocardial injury (TnT/CK-MB/LDH), whereas UPRmt activators suppress these markers. Gene expression analysis showed that the UPRmt could upregulate antioxidant enzymes localized in mitochondria that inhibit mitochondrial ROS generation, which in turn inhibits ischemia-reperfusion-induced myocardial oxidative stress injury. Ameliorating the vulnerability of cardiomyocytes in the ischemia-reperfusion state, the UPRmt also stimulates the transcription of the antiapoptotic proteins Bcl2 and c-IAP.

In addition, Mitotracker-related laser confocal assays showed that UPRmt activation maintained mitochondrial morphology and structural integrity. Notably, administration of a UPRmt antagonist increased myocardial infarct size and cardiac dysfunction in mice. Intervention with UPRmt antagonists also enhanced mitochondrial ROS generation, increased lipid peroxidation levels, and suppressed the transcription of antiapoptotic genes. In animal models of septic cardiomyopathy, significant cardiac ejection dysfunction was observed in the early summer, and oligomycin intervention significantly improved cardiac ejection function. During lipopolysaccharide- (LPS-) induced cardiomyocyte inflammatory injury, severe mitochondrial ROS accumulation, MMP loss, and increased mitochondrial fragmentation occur. Oligomycin can significantly inhibit the overproduction of ROS, restore the mitochondrial membrane potential, and maintain mitochondrial structure and normality. These findings suggest that the UPRmt has a protective effect in the process of myocardial cell injury caused by inflammatory injury.

On the other hand, UPRmt may be associated with myocardial or cardiomyocyte damage or death [84]. Protein kinase R (PKR)-knockout and WT mice were exposed to a pressure overload resulting from transverse aortic constriction. While WT and PKR-knockout mice had similar increases in heart size following lateral aortic constriction, PKR knockout mice had much less lung congestion, stronger left ventricular ejection fraction, better contractility, and significantly reduced myocardial fibrosis than WT mice. PKR knockdown attenuates coarctation-induced tumor necrosis factor *α* expression and decreases cardiac expression of proapoptotic factors (Bax and caspase 3) via double-stranded RNA-dependent protein kinase (PKR) overactivation of eIF2*α*, which promotes cardiomyocyte apoptosis and heart failure. By contrast, PKR deficiency attenuates eIF2*α* overactivation and protects cardiomyocytes [84].

The mitochondrial matrix protease CLPP is the key regulator of the UPRmt [85]. In a study in which CLPP was knocked out in the hearts of mitochondrial aspartyl-tRNA synthetase 2 (DARS2)-deficient mice, the mouse myocardium exhibited an excessive UPRmt due to dysregulation of mitochondrial transcriptional regulation. Unexpectedly, cardiomyocyte mitochondrial dysfunction and reduced mitochondrial respiratory function were alleviated by CLPP loss, resulting in increased levels of oxidative phosphorylation. This seemingly contradictory finding indirectly suggests that the UPRmt is cardioprotective under moderate activity, whereas its overactivity may lead to excessive cardiomyocyte injury. Thus, modest UPRmt activation might be beneficial for the removal or repair of damaged mitochondrial proteins. Excessive UPRmt activation may lead to massive cleavage of mitochondrial proteins [86]. Moreover, the three pathways mediating the UPRmt may have interactive mechanisms and reactions.

The three axes of the UPRmt mentioned above, CHOP, Sirt3/FOXO3a, and ER*α*, act synergistically to reduce dysregulation of protein homeostasis in the mitochondria and myocardial injury from oxidative stress and dysregulated calcium homeostasis. Because of the different targeting mechanisms of the three pathways and the different organelles involved in the regulatory process, the synergistic and antagonistic effects of the UPRmt responses regulated by the three pathways in different cardiomyocyte injury models require further pharmacological experimentation, and in vivo and in vitro experimental models should be used for validation.

## 7. Mitochondrial Quality Control and the UPRmt in Cardiomyocytes

Mitochondria, as the site of cellular aerobic respiration and energy production, are also important organelles that transmit cell death signals. Mitochondrial dysfunction is closely related to the occurrence of stress stimuli such as inflammation, hypoxia, ischemia, and high glucose [87]. In the mitochondria, 95% of the proteins are encoded by nuclear DNA, and after cytoplasmic synthesis, the proteins are transferred via the mitochondrial localization sequence (mitochondrial target sequence) to their functional sites of in the mitochondria [88]. Signal transduction and communication between the nucleus and mitochondria regulate the overall working state of the cell [89]. Various mitochondrial proteins, such as the ETC complex, UCP, mitochondrial dynamic protein, mitochondrial Tom complex, and mPTP, affect the production of mitochondrial ROS, thereby determining the degree of mitochondrial damage in cardiomyocytes [90].

Different mitochondrial quality control mechanisms (including mitophagy, mitochondrial fusion and fission, and mitochondrial biosynthesis) can produce certain regulatory strategies for the mitochondrial stress response [91]. In the stress state, although each part of the mitochondrial quality control pathway is activated independently, the regulation of each part of the mechanism in different pathological and injury models also differs [92]. Different mitochondrial quality control pathways were examined individually. Both UPRmt and mitochondrial quality control are regulatory pathways that maintain the normal function of mitochondria, and there is a certain connection between the two [93]. Understanding how the UPRmt integrates or interacts with various mitochondrial quality control pathways to coordinate mitochondrial homeostasis mechanisms, inhibit cardiomyocyte apoptosis or death, improve mitochondrial stress, and maintain mitochondrial homeostasis is important.

### 7.1. Mitochondrial Autophagy and the UPRmt Response

Mitochondrial autophagy is mainly divided into two types, receptor- and nonreceptor-mediated, and is an important regulatory mechanism for maintaining mitochondrial renewal and function [94]. It plays an important role in regulating the number of mitochondria and is involved in both physiological and pathological processes [95]. Mitophagy is divided into receptor-dependent mitophagy mediated by FUNDC1 and BNIP3, and receptor-independent mitophagy mediated by PINK1/Parkin [3]. Mitophagy and UPRmt are involved in the repair of damaged mitochondria by stimulating responses and protective mechanisms [10]. This study found that both mitophagy pathways can interact with the UPRmt mechanism. A study on myocardial cell inflammatory injury found that FUNDC1-mediated mitophagy can synergize with UPRmt to regulate the cardiomyocyte protection mechanism jointly. Lipopolysaccharide- (LPS-) induced sepsis plays a role in cardiac insufficiency and mitochondrial damage, resulting in excessive accumulation of mitochondrial ROS and significantly reduced TOM20 expression levels [96]. Both the UPRmt activator oligomycin and the mitophagy activator urolithin A significantly reduced LPS-induced cardiac function damage and myocardial cell mitochondrial damage, reversed the excessive accumulation of mitochondrial ROS, increased the expression of TOM20, and restored the mitochondrial membrane potential. FUNDC1 conditional knockout mice counteract the ameliorating effects of the mitophagy activator urolithin A on LPS-induced cardiac function impairment and cardiomyocyte mitochondrial damage [96]. Mitophagy activators had no direct effect on the UPRmt; however, the expression of UPRmt proteins was significantly upregulated after gene knockout of FUNDC1. Knockdown of ATF6 did not affect the protective effect of mitophagy activator on mitochondria but attenuated the protective effect of the UPRmt. Knockdown of FUNDC1 also did not affect mitochondrial protection by the UPRmt activator oligomycin. It has been suggested that FUNDC1 is an important pathway for mitophagy activators to regulate mitophagy and improve mitochondrial inflammatory injury in cardiomyocytes. The mechanisms of action of UPRmt activator oligomycin and mitophagy activator urolithin A on myocardial cell mitochondrial injury are different. During LPS-induced mitochondrial injury in cardiomyocytes, the transcription levels of ATF5, mtDNAj, clpP, LONP1, CHOP, Hsp10, and HSP60 were significantly increased, suggesting that UPRmt is significantly activated [96].

Mitophagy activators can significantly inhibit gene transcription. Interestingly, the UPRmt is further activated after FUNDC1 knockout, and the gene transcription levels are further increased than that after LPS intervention, whereas mitophagy activators were increased after FUNDC1 knockout. Thus, FUNDC1 does not have a regulatory effect on the UPRmt. In a coregulatory mechanism, mitophagy-mediated mitochondrial and cardiomyocyte protection is partially attenuated when the UPRmt is inhibited by ATF6 knockout. Mechanistically, the interaction between endogenous UPRmt and mitophagy can play a compensatory role in maintaining mitochondrial homeostasis. Although activation or inhibition of the UPRmt has no modulating effect on mitophagy, inhibition of the UPRmt impairs mitochondrial function or structure, potentially leading to an overload of the cardioprotective effects of mitophagy.

**Figure 4:**
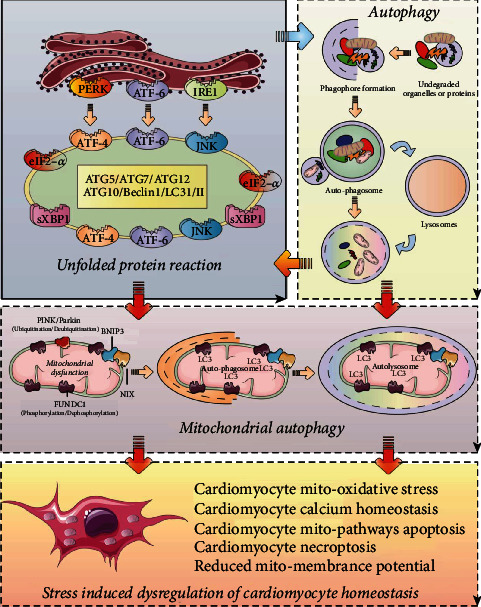
Work together to coordinate cardiomyocytes mitochondrial homeostasis, interaction of UPR with autophagy and mitophagy. The unfolded protein response (UPR) interacts with mitophagy and autophagy. Autophagy can form autophagosomes through the separation membrane initiated by wrapping cytoplasmic components (proteins and organelles) in cardiomyocytes inside the cell. These damaged or redundant proteins and organelles eventually fuse with lysosomes, which in turn are degraded. This process induces the activation of eIF2a and ATF4.Moreover, the accumulation of useless or damaged mitochondria in organelles also removes redundant or dysfunctional mitochondria by activating nonreceptor-mediated (PINK/Parkin) or receptor-mediated (FUNDC1) mitophagy. UPR can also coordinate with FUNDC1-mediated mitophagy under the activated state to jointly protect the mitochondria of cardiomyocytes under inflammation and maintain mitochondrial homeostasis. Mechanistically, deletion of autophagy genes may also induce UPR, suggesting a negative feedback mechanism. When the synergistic effect of mitochondrial UPR and mitophagy is dysregulated, it leads to dysregulation of cardiomyocyte homeostasis, activates cardiomyocyte mitochondrial dysfunction and mediates mitochondrial pathway apoptosis.

As shown in Figure 4, the synergistic effect of mitophagy and the UPRmt is an important part of the mitochondrial protection mechanism in cardiomyocytes. Protein mass and mitochondrial mass are also critical for the regulation of cardiomyocyte viability, and the study also found that the mitochondrial outer membrane protein FUNDC1 can interact with the mitochondrial chaperone HSC70 to promote mitochondrial translocation of unfolded cytoplasmic proteins to degrade or form nonfolded cytoplasmic proteins via LONP1. Aggregate mitochondria-associated protein aggregates [97].

Myocardial ischemia-reperfusion injury can inhibit the expression of LONP1, mtDNAj, clpP, and HSP10, induce excessive accumulation of mitochondrial ROS, and increase the level of mitochondrial fragmentation, leading to dysregulation of mitochondrial homeostasis in cardiomyocytes and activation of cardiomyocyte apoptotic pathways. The expression levels of myocardial CM-KB, TnT, and LDH further increased with injury to ejection function. Under oligomycin treatment, the transcription levels of LonpP1, mtDNAj, clpP, and Hsp10 were further increased in cardiomyocytes in an ischemia-reperfusion state, and the excessive accumulation of mitochondrial ROS and mitochondrial fragmentation were also inhibited. The expression levels of TnT and LDH were further inhibited and the apoptotic pathway was blocked [83].

Ischemia-reperfusion injury can inhibit the expression of LONP1, mtDNAj, clpP, and Hsp10 in cardiac-specific FUNDC1 conditional knockout cells to a greater extent than in cardiomyocytes without conditional knockout of FUNDC1 [83].

After further shRNA-mediated Parkin knockout, significant expression of the UPRmt mRNA marker in cardiomyocytes was induced. After shRNA-mediated FUNDC1 knockout, the expression of the UPRmt mRNA marker in cardiomyocytes was not significant, although both Parkin^OE^ and UPRmt mRNA marker expression appeared to be increased. These results suggest that UPRmt activation in the state of ischemia-reperfusion injury is dependent on different types of mitophagy (receptor- and nonreceptor-mediated) and can be differentially regulated by distinct mechanisms; however, the different regulatory mechanisms and whether they are related to the cell model and the time conditions of ischemia-reperfusion remain to be experimentally verified [83].

Within the mitochondrial matrix, PINK1/Parkin mediates receptor-independent mitophagy to alleviate protein toxicity [98]. Mitophagy can eliminate protein aggregates within the mitochondria during mitochondrial homeostasis. PINK1 recruits parkin to the mitochondrial subdomains following mitochondrial proteotoxic injury. Parkin then colocalizes with LC3 on polarized mitochondria that contain misfolded proteins. Compared to the inhibition of Drp1-mediated mitochondrial fission, knockdown of Drp1 enhances Parkin recruitment to fused mitochondria and increases mitophagy levels [98].

These results verify that PINK1/Parkin-mediated receptor-independent mitophagy can inhibit mitochondrial proteotoxicity, maintain mitochondrial protein homeostasis, and is also associated with Drp1-mediated mitochondrial fusion/fission [99]. The mechanism of PINK1-mediated receptor-independent mitophagy also showed that under stress conditions, the myocardial line UPRmt is enhanced, and this process is regulated by the PINK1 mitophagy pathway. Studies have found that mitochondria-specific chaperones and proteases can inhibit the synthesis of misfolded proteins, regulate mitochondrial fission/fusion, and regulate mitophagy to maintain mitochondrial homeostasis at the organelle level. Excessive accumulation of unfolded proteins can promote the accumulation of PINK1, which can also lead to the mitochondrial translocation of PARK2 and nonreceptor-mediated mitophagy. By knocking down the LONP1 protease, the accumulation of PINK1 can be enhanced to a certain level. In the regulation mechanism of mitophagy and the UPRmt, we believe that the accumulation of unfolded proteins in the mitochondria can trigger mitophagy, and the two not only cooperate to regulate and protect mitochondria but can also coordinate with each other. When the level of phagocytosis decreases, the load level of the UPRmt increases, and vice versa, so that the two can coordinate the protective mechanisms of cardiomyocytes [99].

Both the mitochondrial UPR and mitophagy are specific repair pathways within the mitochondria of cardiomyocytes. The UPRmt can maintain mitochondrial internal protein homeostasis and normalization through mitochondrial protein folding and degradation, preventing abnormal protein accumulation in the mitochondria [100].

Mitophagy can selectively remove damaged mitochondria via receptor-dependent or receptor-independent pathways. Unlike mitophagy, the UPRmt can dynamically control the import or export of mitochondrial proteins and affect mitochondrial energy metabolism [101]. Mitophagy and the UPRmt are similar in that both require moderate activation under stress, and if overactivated, can lead to mitochondrial dysfunction and activation of the mitochondrial pathway of apoptosis [102, 103]. Therefore, mitophagy and the UPRmt within a limited range maintain the cardioprotective effects of mitochondrial mass, and the balance and synergistic protective effects of the two are indispensable in mitochondrial protein homeostasis.

### 7.2. Mitochondrial Fission and the UPRmt

Mitochondria are centers of energy metabolism in cells and are dynamic organelles [104]. Mitochondrial dynamics are an important way to regulate the balance mechanism of mitochondrial fusion and fission [105]. The synergistic effect of mitochondrial fusion and fission is essential for maintaining normal mitochondrial morphology and function [106]. Dysregulation of mitochondrial fission may induce an increase in mitochondrial unfolded proteins, affecting mitochondrial proteostasis, and can further lead to dysregulation of mitochondrial homeostasis and energy metabolism [107].

Mitochondrial dynamics are inextricably linked to the development of mitochondrial damage and the UPRmt in cardiomyocytes [108]. During stress injury, such as ischemia or inflammation, mitochondrial fission is specifically increased and mitochondrial fusion is inhibited [109]. An imbalance between mitochondrial fission and fusion is associated with ischemia/reperfusion-induced cardiac dysfunction or cardiomyocyte injury. Mitochondrial fission is mediated by Drp1 and the mitochondrial outer membrane-anchored adaptor proteins Fis1 and Mff [110]. Mitochondrial fusion is mediated by external Mfn1 and Mfn2 on the membrane and regulated by OPA1 on the inner membrane [111]. Mitochondrial fusion allows normal mitochondria to combine with unfolded proteins of partially damaged mitochondria to reduce the stress response; mitochondrial fission is required for the formation of new mitochondria, while also separating damaged mitochondria to achieve mitochondrial quality and quantity control [112].

Mitochondrial fission is mainly divided into three steps: Drp1 protein is transferred from the cytoplasm to the mitochondria; the endoplasmic reticulum and actin cooperate to drive Drp1 protein contraction, and the Drp1 protein further shrinks until mitochondrial fission occurs. Mitochondria with inhibited mitochondrial fusion may undergo mitochondrial fission, leading to the accumulation of large amounts of unstable unfolded proteins, thus activating the UPRmt, which in turn inhibits mitochondrial dysfunction caused by abnormal mitochondrial dynamics [113]. Related research on the regulatory ability of proteases and the ubiquitin-proteasome system showed that proteases have a certain connection with the dynamic changes in mitochondria [114, 115]. The ETC in mitochondria is a source of ROS that destroys proteins. Mitochondrial dysfunction is associated with pathological conditions and is thus an important hallmark of cardiomyocyte injury, which is associated with the failure of mitochondrial proteostasis.

### 7.3. Mitochondrial Fusion and the UPRmt

In addition to pathological mitochondrial fission, stress-induced mitochondrial fusion dysfunction is also an important cause of dysregulated mitochondrial proteostasis and the UPR response. Although mitochondria form a compartment unique to the proteasome, multiple lines of evidence suggest that cytoplasmic UPRmt plays a crucial role in the quality control of mitochondrial proteins. Proteasomes affect mitochondrial proteins during biogenesis and maturation. The role of the UPRmt goes beyond removing damaged proteins; it also modulates mitochondrial proteome composition, modulates organelle dynamics, and protects cellular homeostasis from mitochondrial exhaustion [116]. The domains of several key effectors (Mfn1 and Mfn2) and fission proteins (Fis1 and Drp1) of mitochondrial fusion are exposed on the cytoplasmic side of the mitochondrial membrane [117].

Ischemia-reperfusion can increase the expression of mitochondrial fission kinases in Drp1/Fis1/MFF, which in turn leads to the inhibition of the expression of OPA1, Mfn1, and Mfn2, increase in mitochondrial fragmentation level, and considerable shortening of mitochondrial length [118]. The UPRmt activator oligomycin can increase the expression levels of OPA1, Mfn1, and Mfn2 to different degrees, inhibit mitochondrial fragmentation, and increase mitochondrial length [119]. Inhibitors of the UPRmt can reverse this phenomenon, leading to increased levels of mitochondrial fission. UPRmt activation may affect mitochondrial dynamics. Under conditions of increased mitochondrial fission, the UPRmt can maintain the stability of the mitochondrial structure [120]. Stress-induced processing of OPA1 by OMA1 completely converts OPA1 into a short isoform, inhibits fusion, and triggers mitochondrial fission. Specific proteases in the UPRmt directly participate in the regulation of mitochondrial dynamics by cleaving OPA1 and balancing mitochondrial fission and fusion [121]. ClpP is an endopeptidase component of the mitochondrial matrix and an important regulator of the UPRmt. ClpP-deficient cells can undergo extensive mitochondrial structural changes and mitochondrial dysfunction [122].

If the mitochondrial electron transport chain is dysfunctional, the activity of mitochondrial respiratory chain (ETC) complexes I and II is reduced, mitochondrial basal respiration is inhibited, and the oxygen consumption rate is affected to some extent. Knockdown of ClpP further affects mitochondrial dynamics, increases the expression of the Drp1, and inhibits UPRmt sensing, which further results in increased ROS production and decreased membrane potential. ClpP knockdown inhibited the protein expression level of PGC1a and increased the phosphorylation of eIF2*α*, suggesting that ClpP deficiency has an impact on the UPRmt and can also lead to dysregulation of mitochondrial dynamics and increased levels of mitochondrial fission. ClpP can protect mitochondrial mass by altering mitochondrial dynamics [123].

### 7.4. Mitochondrial Biosynthesis Machinery and the UPRmt

**Figure 5:**
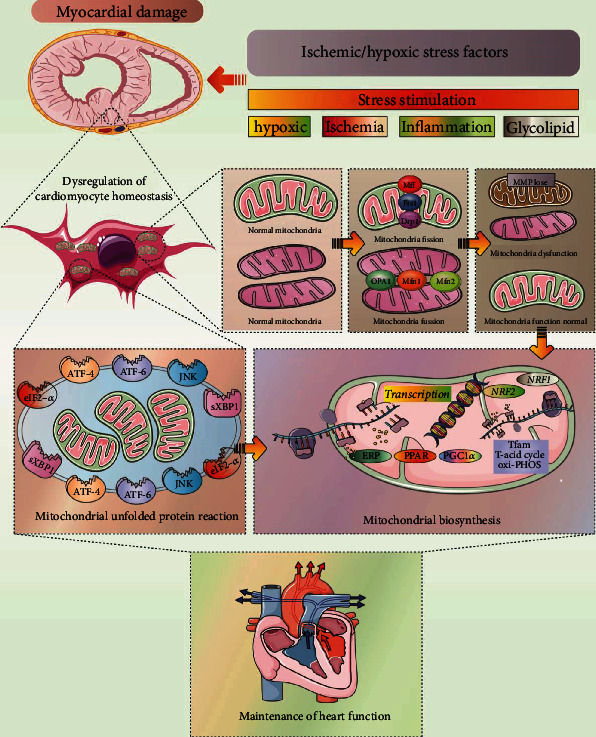
Stress factor-induced UPRmt is blocked and mitochondrial dynamics and biosynthesis dysfunction. Under the influence of stress factors, mitochondrial quality control can synergize with UPRmt. Hypoxia/ischemia, inflammation and hyperglycemia/hyperlipidemia induce mitochondrial dysfunction, which in turn leads to the activation of mitochondrial fission kinesins MFF/Fis1/Drp1 and the inhibition of the expression of mitochondrial fusion proteins OPA1/Mfn1 and Mfn2. This in turn leads to aggravated mitochondrial fission and mitochondrial dysfunction. This further leads to PGC1a and NRF1/NRF2-mediated mitochondrial biosynthesis impairment, decreased Tfam and mitochondrial transcription levels, and disturbance of the tricarboxylic acid cycle and oxidative phosphorylation levels, which may be important pathways of mitochondrial damage. At this time, UPR is activated, which may indirectly compensate for mitochondrial biosynthesis dysfunction and maintain cardiomyocyte function and cardiac function.

As shown in Figure 5, the mitochondrial biosynthetic machinery and UPRmt are also closely linked. Mitochondrial biosynthesis is the formation of new mitochondria to maintain and restore mitochondrial structure and quantity [124]. Mitochondrial biosynthesis-related disorders are closely related to a variety of diseases, including cardiomyopathy and degenerative diseases [125]. It is currently known that PGC1*α* and TFAM are involved in the regulation of mitochondrial biosynthesis. Active components of natural medicines, such as quercetin, ginsenosides, flavonoids, and polysaccharides, can also improve mitochondrial biosynthesis through different pathways [91, 126]. Sirt3 can deacetylate FOXO3 to inhibit mitochondrial oxidative stress. Sirt3-mediated deacetylation of FOXO3 can also increase the protein expression of mitochondrial PGC1a and TFAM, regulate mitochondrial biosynthesis, and improve mitochondrial energy metabolism. Moreover, under the influence of SIRT3-mediated FOXO3, BNIP3-mediated mitophagy, and Drp1/Fis1-mediated mitochondrial fusion/fission are further regulated. The deacetylation of FOXO3 by SIRT3 may be a mechanism that affects the level of mitochondrial biosynthesis by regulating mitophagy and mitochondrial fusion/fission, and maintaining the level of mitochondrial energy metabolism and mitochondrial homeostasis [126].

Selective activation of the UPRmt can also induce increased transcription levels of Sirt3, further enhancing PGC1a-mediated mitochondrial biosynthesis [127]. A study on hypoxic cardiomyocytes found that hypoxia can further inhibit the transcription level of PGC1a and TFAM, suggesting that mitochondrial biosynthesis is inhibited under stress, whereas a mitochondrial unfolded protein activator can increase PGC1a/TFAM under hypoxia. The transcription level of TFAM maintains the stability of mitochondrial DNA, whereas a mitochondrial unfolded protein inhibitor can inhibit the transcription of PGC1a/TFAM. These results suggest that the UPRmt can affect mitochondrial biosynthesis [83].

Another study found that a decrease in CHOP can lead to decreased expression of nuclear-encoded cytochrome c oxidase IV. Chronic contractile activity can prevent the decrease in cytochrome c oxidase IV caused by CHOP deficiency, increase Sirt3 and cpn10, and increase mitochondrial biogenesis in myotubes by two-three times. The UPRmt regulates mitochondrial biogenesis and improves mitochondrial function [83]. Mitochondrial biosynthesis, as an important linking mechanism between mitochondrial quality control and the UPRmt, can affect the synthesis or damage of mtDNA [128], thereby affecting the activity of cardiomyocytes. However, there are few studies on the interaction mechanism between mitochondrial biosynthesis and the mitochondrial unfolded protein reaction. In the future, more models may be developed to verify and better explain the mechanism of mitochondrial synthesis.

## 8. Summary and Outlook

Mitochondrial protein homeostasis plays a key regulatory role in cell survival and mitochondrial pathophysiological mechanisms, and mitochondrial unfolded proteins are the upstream regulatory nodes for maintaining mitochondrial function and mitochondrial energy metabolism. The UPRmt can regulate mitochondrial structure, quality, and quantity through multiple pathways, including PGC1a/Tfam/Nrf1/2-mediated mitochondrial biogenesis, FUNDC1/BNIP3-mediated receptor-dependent mitophagy, PINK/Parkin-mediated receptor-independent mitochondrial self-digestion, Drp1/Fis1/OPA1/Mfn1/Mfn2-mediated mitochondrial fusion and fission, and other functions showing adaptive changes, thereby maintaining a stable and healthy mitochondrial pool and improving cardiomyocyte viability.

Whether under physiological conditions or under stress conditions caused by various factors, each process of mitochondrial quality control and UPRmt change is interactive and interdependent, and maintaining a balanced relationship between the UPRmt and the regulation of mitochondrial quality control mechanisms is particularly important. As an important mitochondrial protein regulation mechanism, the UPRmt regulates mitochondrial function by mediating UPRmt. Our current research mostly focuses on in vitro experiments, and there are relatively few studies on the mechanism by which the UPRmt regulates mitochondrial quality control in animal research. What is worth paying attention to in the future is that the related regulatory pathways and molecular mechanisms of UPRmt are relatively complex; for example, PGC1a can both regulate mitochondrial biosynthesis and affect the UPRmt. Whether there is an interaction between the UPRmt and various signal transduction pathways related to mitochondrial quality control should be determined.

Mitophagy, mitochondrial fusion/fission, and the UPRmt have balanced mechanisms. Excessive activation or decreased regulation can directly affect mitochondrial biosynthesis, resulting in mitochondrial dysfunction. Moderate UPRmt activation, mitophagy, and mitochondrial dynamics are beneficial for maintaining mitochondrial protein homeostasis with cardioprotective effects. However, UPRmt hyperactivation, mitophagy, and mitochondrial dynamics may also exacerbate mitochondrial dysfunction and aggravate cardiomyocyte injury. How the specific mechanism is regulated, whether there are different models, the influence of stress stimuli, and the activation or inhibition of the above mechanisms have different levels of influence. In the future, this also needs to be verified and explained using different models.

The functional status of mitochondria in cardiomyocytes often determines their fate, and investigation of the role of mitochondria in cardiovascular diseases has become a research hotspot. The UPRmt is a protective factor for cardiomyocytes and may be an important target for development of cardioprotective treatment strategies in cardiovascular diseases. Because the understanding of UPRmt is still not sufficiently comprehensive, there are relatively few related therapeutic drug studies and more research is needed in the future. Further research is needed to provide strong evidence for the clinical translation of UPRmt-based therapies and the treatment of related cardiovascular diseases. Drug research should also focus on determining the dose of drug intervention, the time course of drug intervention, and whether drugs have conflicting effects on mitochondrial quality monitoring and the UPRmt.

Mitochondria are small organelles that transmit cellular metabolic and death signals. Mitochondrial energy metabolism disorder is closely related to mitochondrial protein homeostasis and the mitochondrial UPR response. Signal transduction and communication between the nucleus and mitochondria regulate the overall energy metabolism of the cells. The unique UPRmt pathway and the mechanism induced by the abnormal structure of mitochondrial proteins have been gradually elucidated, revealing that the UPRmt pathway plays an important role in the regulation of mitochondrial energy metabolism. Due to the complexity and diversity of mitochondrial functions, there are multiple UPRmt pathways and multiple regulatory factors in mammalian cells, which can directly or indirectly regulate mitochondrial energy metabolism and mitochondrial homeostasis.

Mitochondrial oxidative stress (OS), mitochondrial calcium homeostasis, oxidative phosphorylation, and tricarboxylic acid cycle reactions can all affect mitochondrial protein homeostasis to varying degrees. Stress injury leads to mitochondrial metabolic dysfunction, which affects the UPR response. This causes a vicious cycle of increased oxidative stress damage, calcium overload, and mitochondrial respiratory dysfunction, induces mitochondrial membrane permeability transition pore opening and increased sensitivity to oxidative stress, leading to aggravated mitochondrial dysfunction and induction of apoptosis. The UPRmt is both the site and the target of mitochondrial energy metabolism damage. Since mitochondrial biosynthesis depends on the coordinated action of all mitochondrial synthesis components, this process is coordinated by PGC-1*α* to activate nuclear respiratory factors 1 and 2 (NRF-1 and NRF-2), enhancing nuclear-encoded mitochondrial transcription factors. Transcription factor A (TFAM) binds to mitochondrial DNA, initiates mitochondrial transcription and genome replication, and ultimately leads to reduced mitochondrial biosynthesis, regardless of UPRmt dysfunction or impairment of mitochondrial respiratory chain function. Therefore, future research should focus on restoring mitochondrial energy metabolism while regulating the UPRmt, which is also an important target mechanism for maintaining mitochondrial homeostasis in cardiomyocytes.

Moreover, the UPRmt can play multiple protective roles or damage mechanisms in the physiological and pathological processes of cardiomyocytes. Therefore, as a “double-edged sword,” it is very important in the maintenance of mitochondrial protein homeostasis and intracellular environmental homeostasis of cardiomyocytes. First, the UPRmt can protect mitochondrial function after myocardial infarction and can promote the clearance and degradation of damaged mitochondrial unfolded proteins. Future research on the intervention mechanism of targeted drugs should focus on elucidating how drugs affect this process and ensuring that mitochondrial proteins are normally degraded into components of basal cell internal circulation to support the energy metabolism function of cardiomyocytes under stress conditions.

In addition, the mechanism of action of the UPRmt on mitochondrial biogenesis under stress in cardiomyocytes has not been fully elucidated. Mild spiciness can be further studied through relevant mechanisms to provide a targeted drug research basis for promoting mitochondrial self-renewal and regeneration.

Most studies on cardiomyocyte damage and mitochondria-related mechanisms suggest that mitophagy serves as a “Guardian” of mitochondrial function and cardiomyocyte stability. UPRmt overexpression, which promotes damage and mitochondrial protein homeostasis, also leads to reverse regulation of cardioprotective effects. Moreover, it is noteworthy that the interaction mechanism and complementary role of the UPRmt and mitophagy need to be discussed further. The extent of the UPRmt and mitophagy also depend on the severity and duration of stress injury in cardiomyocytes. Therefore, the “double-edged sword” of the UPRmt needs to be reasonably utilized. In future drug and pharmacological research, the interaction mechanisms of mitochondrial dynamics, mitochondrial autophagy, and mitochondrial biosynthesis should be investigated as much as possible to better lay a preliminary foundation for the study of mitochondrial quality control and the steady-state mechanisms of cardiomyocytes.

## Data Availability

The data used to support the findings of this study are available from the corresponding author upon request.

## References

[1] Zimetbaum P. J., Josephson M. E. (2003). Use of the electrocardiogram in acute myocardial infarction. *The New England Journal of Medicine*.

[2] Jacoby R. M., Nesto R. W. (1992). Acute myocardial infarction in the diabetic patient: pathophysiology, clinical course and prognosis. *Journal of the American College of Cardiology*.

[3] Cai C., Guo Z., Chang X. (2022). Empagliflozin attenuates cardiac microvascular ischemia/reperfusion through activating the AMPK*α*1/ULK1/FUNDC1/mitophagy pathway. *Redox Biology*.

[4] Yu H., Zhang F., Yan P. (2021). LARP7 protects against heart failure by enhancing mitochondrial biogenesis. *Circulation*.

[5] Gibb A. A., Lazaropoulos M. P., Elrod J. W. (2020). Myofibroblasts and fibrosis: mitochondrial and metabolic control of cellular differentiation. *Circulation Research*.

[6] Münch C., Harper J. W. (2016). Mitochondrial unfolded protein response controls matrix pre-RNA processing and translation. *Nature*.

[7] Zhou H., Ren J., Toan S., Mui D. (2021). Role of mitochondrial quality surveillance in myocardial infarction: from bench to bedside. *Ageing Research Reviews*.

[8] Lemberg M. K., Strisovsky K. (2021). Maintenance of organellar protein homeostasis by ER-associated degradation and related mechanisms. *Molecular Cell*.

[9] Hoppe T., Cohen E. (2020). Organismal protein homeostasis mechanisms. *Genetics*.

[10] Wang Y., Jasper H., Toan S., Muid D., Chang X., Zhou H. (2021). Mitophagy coordinates the mitochondrial unfolded protein response to attenuate inflammation-mediated myocardial injury. *Redox Biology*.

[11] Zhang Q., Wu X., Chen P. (2018). The mitochondrial unfolded protein response is mediated cell-non-autonomously by Retromer-dependent Wnt signaling. *Cell*.

[12] Zhu H., Toan S., Mui D., Zhou H. (2021). Mitochondrial quality surveillance as a therapeutic target in myocardial infarction. *Acta Physiologica*.

[13] Chang X., Zhang T., Liu D. (2021). Puerarin attenuates LPS-induced inflammatory responses and oxidative stress injury in human umbilical vein endothelial cells through mitochondrial quality control. *Oxidative Medicine and Cellular Longevity*.

[14] Chang X., Zhang T., Meng Q. (2021). Quercetin improves cardiomyocyte vulnerability to hypoxia by regulating SIRT1/TMBIM6-related mitophagy and endoplasmic reticulum stress. *Oxidative Medicine and Cellular Longevity*.

[15] Xu M., Xue R. Q., Lu Y. (2019). Choline ameliorates cardiac hypertrophy by regulating metabolic remodelling and UPRmt through SIRT3-AMPK pathway. *Cardiovascular Research*.

[16] Sorrentino V., Menzies K. J., Auwerx J. (2018). Repairing mitochondrial dysfunction in disease. *Annual Review of Pharmacology and Toxicology*.

[17] Spinelli J. B., Haigis M. C. (2018). The multifaceted contributions of mitochondria to cellular metabolism. *Nature Cell Biology*.

[18] Merry T. L., Chan A., Woodhead J. S. T. (2020). Mitochondrial-derived peptides in energy metabolism. *American Journal of Physiology Endocrinology and Metabolism*.

[19] Schapira A. H. (2012). Mitochondrial diseases. *The Lancet*.

[20] Chang X., Yao S., Wu Q., Wang Y., Liu J., Liu R. (2021). Tongyang Huoxue decoction (TYHX) ameliorating hypoxia/reoxygenation-induced disequilibrium of calcium homeostasis and redox imbalance via regulating mitochondrial quality control in sinoatrial node cells. *Oxidative Medicine and Cellular Longevity*.

[21] Vafai S. B., Mootha V. K. (2012). Mitochondrial disorders as windows into an ancient organelle. *Nature*.

[22] Chacinska A., Koehler C. M., Milenkovic D., Lithgow T., Pfanner N. (2009). Importing mitochondrial proteins: machineries and mechanisms. *Cell*.

[23] Wiedemann N., Pfanner N. (2017). Mitochondrial machineries for protein import and assembly. *Annual Review of Biochemistry*.

[24] Priesnitz C., Becker T. (2018). Pathways to balance mitochondrial translation and protein import. *Genes & Development*.

[25] Chang X., Zhang T., Wang J. (2021). SIRT5-related desuccinylation modification contributes to quercetin-induced protection against heart failure and high-glucose-prompted cardiomyocytes injured through regulation of mitochondrial quality surveillance. *Oxidative Medicine and Cellular Longevity*.

[26] Chang X., Zhang W., Zhao Z. (2020). Regulation of mitochondrial quality control by natural drugs in the treatment of cardiovascular diseases: potential and advantages. *Frontiers in Cell and Developmental Biology*.

[27] Wolf C., López Del Amo V., Arndt S. (2020). Redox modifications of proteins of the mitochondrial fusion and fission machinery. *Cell*.

[28] Zhu L., Luo X., Fu N., Chen L. (2021). Mitochondrial unfolded protein response: a novel pathway in metabolism and immunity. *Pharmacological Research*.

[29] Zhu L., Zhou Q., He L., Chen L. (2021). Mitochondrial unfolded protein response: an emerging pathway in human diseases. *Free Radical Biology & Medicine*.

[30] Senft D., Ronai Z. A. (2015). UPR, autophagy, and mitochondria crosstalk underlies the ER stress response. *Trends in Biochemical Sciences*.

[31] Weng H., Ma Y., Chen L. (2020). A new vision of mitochondrial unfolded protein response to the Sirtuin family. *Current Neuropharmacology*.

[32] Seli E., Wang T., Horvath T. L. (2019). Mitochondrial unfolded protein response: a stress response with implications for fertility and reproductive aging. *Fertility and Sterility*.

[33] Smyrnias I. (2021). The mitochondrial unfolded protein response and its diverse roles in cellular stress. *The International Journal of Biochemistry & Cell Biology*.

[34] Shen G., Liu W., Xu L., Wang L. L. (2020). Mitochondrial unfolded protein response and its roles in stem cells. *Stem Cells and Development*.

[35] Naresh N. U., Haynes C. M. (2019). Signaling and regulation of the mitochondrial unfolded protein response. *Cold Spring Harbor Perspectives in Biology*.

[36] Jenkins E. C., Chattopadhyay M., Germain D. (2021). Folding mitochondrial-mediated cytosolic proteostasis into the mitochondrial unfolded protein response. *Frontiers in Cell and Developmental Biology*.

[37] Vögtle F. N. (2021). Open questions on the mitochondrial unfolded protein response. *The FEBS Journal*.

[38] Jiang D., Cui H., Xie N. (2020). ATF4 mediates mitochondrial unfolded protein response in alveolar epithelial cells. *American Journal of Respiratory Cell and Molecular Biology*.

[39] Okamoto T., Yamamoto H., Kudo I. (2017). HSP60 possesses a GTPase activity and mediates protein folding with HSP10. *Scientific Reports*.

[40] Melber A., Haynes C. M. (2018). UPR^mt^ regulation and output: a stress response mediated by mitochondrial-nuclear communication. *Cell Research*.

[41] Deng P., Haynes C. M. (2017). Mitochondrial dysfunction in cancer: potential roles of ATF5 and the mitochondrial UPR. *Seminars in Cancer Biology*.

[42] Zhang J., Xiang H., Liu J., Chen Y., He R. R., Liu B. (2020). Mitochondrial Sirtuin 3: new emerging biological function and therapeutic target. *Theranostics*.

[43] Papa L., Germain D. (2014). SirT3 regulates the mitochondrial unfolded protein response. *Molecular and Cellular Biology*.

[44] Barros R. P., Gustafsson J. (2011). Estrogen receptors and the metabolic network. *Cell Metabolism*.

[45] Tait S. W., Green D. R. (2010). Mitochondria and cell death: outer membrane permeabilization and beyond. *Nature Reviews Molecular Cell Biology*.

[46] Madreiter-Sokolowski C. T., Thomas C., Ristow M. (2020). Interrelation between ROS and Ca^2+^ in aging and age- related diseases. *Redox Biology*.

[47] Hara Y., Waters E. M., McEwen B. S., Morrison J. H. (2015). Estrogen effects on cognitive and synaptic health over the lifecourse. *Physiological Reviews*.

[48] Kenny T. C., Germain D. (2017). mtDNA, metastasis, and the mitochondrial unfolded protein response (UPRmt). *Frontiers in Cell and Developmental Biology*.

[49] Finka A., Mattoo R. U., Goloubinoff P. (2016). Experimental milestones in the discovery of molecular chaperones as polypeptide unfolding enzymes. *Annual Review of Biochemistry*.

[50] Ngo J. K., Pomatto L. C., Davies K. J. (2013). Upregulation of the mitochondrial Lon protease allows adaptation to acute oxidative stress but dysregulation is associated with chronic stress, disease, and aging. *Redox Biology*.

[51] Zhang X. P., Glaser E. (2002). Interaction of plant mitochondrial and chloroplast signal peptides with the Hsp70 molecular chaperone. *Trends in Plant Science*.

[52] Biebl M. M., Buchner J. (2019). Structure, function, and regulation of the Hsp90 machinery. *Cold Spring Harbor Perspectives in Biology*.

[53] Park S. W., Ozcan U. (2013). Potential for therapeutic manipulation of the UPR in disease. *Seminars in Immunopathology*.

[54] Maciel L., de Oliveira D. F., Monnerat G., Campos de Carvalho A. C., Nascimento J. H. M. (2020). Exogenous 10 kDa-heat shock protein preserves mitochondrial function after hypoxia/reoxygenation. *Frontiers in Pharmacology*.

[55] Gomez-Pastor R., Burchfiel E. T., Thiele D. J. (2018). Regulation of heat shock transcription factors and their roles in physiology and disease. *Nature Reviews Molecular Cell Biology*.

[56] Ohama N., Sato H., Shinozaki K., Yamaguchi-Shinozaki K. (2017). Transcriptional regulatory network of plant heat stress response. *Trends in Plant Science*.

[57] Germain D. (2016). Sirtuins and the estrogen receptor as regulators of the mammalian mitochondrial UPR in cancer and aging. *Advances in Cancer Research*.

[58] Fan P., Jordan V. C. (2022). PERK, beyond an unfolded protein response sensor in estrogen-induced apoptosis in endocrine-resistant breast cancer. *Molecular Cancer Research : MCR*.

[59] Fan P., Jordan V. C. (2022). Estrogen receptor and the unfolded protein response: double-edged swords in therapy for estrogen receptor-positive breast cancer. *Targeted Oncology*.

[60] Hill S., Sataranatarajan K., Van Remmen H. (2018). Role of signaling molecules in mitochondrial stress response. *Frontiers in Genetics*.

[61] Lepretti M., Martucciello S., Burgos Aceves M. A., Putti R., Lionetti L. (2018). Omega-3 fatty acids and insulin resistance: focus on the regulation of mitochondria and endoplasmic reticulum stress. *Nutrients*.

[62] Shpilka T., Haynes C. M. (2018). The mitochondrial UPR: mechanisms, physiological functions and implications in ageing. *Nature Reviews Molecular Cell Biology*.

[63] Quirós P. M., Prado M. A., Zamboni N. (2017). Multi-omics analysis identifies ATF4 as a key regulator of the mitochondrial stress response in mammals. *The Journal of Cell Biology*.

[64] Fiorese C. J., Schulz A. M., Lin Y. F., Rosin N., Pellegrino M. W., Haynes C. M. (2016). The transcription factor ATF5 mediates a mammalian mitochondrial UPR. *Current Biology : CB*.

[65] Keerthiga R., Pei D. S., Fu A. (2021). Mitochondrial dysfunction, UPRmt signaling, and targeted therapy in metastasis tumor. *Cell & Bioscience*.

[66] Zhao Q., Wang J., Levichkin I. V., Stasinopoulos S., Ryan M. T., Hoogenraad N. J. (2002). A mitochondrial specific stress response in mammalian cells. *The EMBO Journal*.

[67] Kenny T. C., Germain D. (2017). From discovery of the CHOP axis and targeting ClpP to the identification of additional axes of the UPRmt driven by the estrogen receptor and SIRT3. *Journal of Bioenergetics and Biomembranes*.

[68] Malhi H., Kaufman R. J. (2011). Endoplasmic reticulum stress in liver disease. *Journal of Hepatology*.

[69] Chapple S. J., Cheng X., Mann G. E. (2013). Effects of 4-hydroxynonenal on vascular endothelial and smooth muscle cell redox signaling and function in health and disease. *Redox Biology*.

[70] Sun W., Liu C., Chen Q., Liu N., Yan Y., Liu B. (2018). SIRT3: a new regulator of cardiovascular diseases. *Oxidative Medicine and Cellular Longevity*.

[71] He X., Zeng H., Chen J. X. (2019). Emerging role of SIRT3 in endothelial metabolism, angiogenesis, and cardiovascular disease. *Journal of Cellular Physiology*.

[72] Chen J., Chen S., Zhang B., Liu J. (2021). SIRT3 as a potential therapeutic target for heart failure. *Pharmacological Research*.

[73] Rolland S. G., Schneid S., Schwarz M. (2019). Compromised mitochondrial protein import acts as a signal for UPR^mt^. *Cell Reports*.

[74] Yu W., Gao B., Li N. (2017). Sirt3 deficiency exacerbates diabetic cardiac dysfunction: role of Foxo3A-Parkin-mediated mitophagy. *Biochimica et Biophysica acta Molecular Basis of Disease*.

[75] Zheng Y., Shi B., Ma M., Wu X., Lin X. (2019). The novel relationship between Sirt3 and autophagy in myocardial ischemia-reperfusion. *Journal of Cellular Physiology*.

[76] Das D. K., Mukherjee S., Ray D. (2010). Resveratrol and red wine, healthy heart and longevity. *Heart Failure Reviews*.

[77] Kwon S., Kim E. J. E., Lee S. V. (2018). Mitochondria-mediated defense mechanisms against pathogens in caenorhabditis elegans. *BMB Reports*.

[78] Ng M. Y. W., Wai T., Simonsen A. (2021). Quality control of the mitochondrion. *Developmental Cell*.

[79] Anderson N. S., Haynes C. M. (2020). Folding the mitochondrial UPR into the integrated stress response. *Trends in Cell Biology*.

[80] Deng P., Uma Naresh N., Du Y. (2019). Mitochondrial UPR repression duringPseudomonas aeruginosainfection requires the bZIP protein ZIP-3. *Proceedings of the National Academy of Sciences of the United States of America*.

[81] Lin K. M., Lin B., Lian I. Y., Mestril R., Scheffler I. E., Dillmann W. H. (2001). Combined and individual mitochondrial HSP60 and HSP10 expression in cardiac myocytes protects mitochondrial function and prevents apoptotic cell deaths induced by simulated ischemia-reoxygenation. *Circulation*.

[82] Tian Y., Garcia G., Bian Q. (2016). Mitochondrial stress induces chromatin reorganization to promote longevity and UPR(mt). *Cell*.

[83] Wang Y. T., Lim Y., McCall M. N. (2019). Cardioprotection by the mitochondrial unfolded protein response requires ATF5. *American Journal of Physiology Heart and Circulatory Physiology*.

[84] He X., Zeng H., Chen J. X. (2016). Ablation of SIRT3 causes coronary microvascular dysfunction and impairs cardiac recovery post myocardial ischemia. *International Journal of Cardiology*.

[85] Ren J., Bi Y., Sowers J. R., Hetz C., Zhang Y. (2021). Endoplasmic reticulum stress and unfolded protein response in cardiovascular diseases. *Nature Reviews Cardiology*.

[86] Fernández A., Ordóñez R., Reiter R. J., González-Gallego J., Mauriz J. L. (2015). Melatonin and endoplasmic reticulum stress: relation to autophagy and apoptosis. *Journal of Pineal Research*.

[87] Ji H., Wang J., Muid D., Song W., Jiang Y., Zhou H. (2022). FUNDC1 activates the mitochondrial unfolded protein response to preserve mitochondrial quality control in cardiac ischemia/reperfusion injury. *Cellular Signalling*.

[88] Wang H., Xu X., Fassett J. (2014). Double-stranded RNA-dependent protein kinase deficiency protects the heart from systolic overload-induced congestive heart failure. *Circulation*.

[89] Seiferling D., Szczepanowska K., Becker C. (2016). Loss of CLPP alleviates mitochondrial cardiomyopathy without affecting the mammalian UPRmt. *EMBO Reports*.

[90] Matilainen O., Quirós P. M., Auwerx J. (2017). Mitochondria and epigenetics - crosstalk in homeostasis and stress. *Trends in Cell Biology*.

[91] Chang X., Lochner A., Wang H. H. (2021). Coronary microvascular injury in myocardial infarction: perception and knowledge for mitochondrial quality control. *Theranostics*.

[92] Wang S., Zhu H., Li R. (2022). DNA-PKcs interacts with and phosphorylates Fis1 to induce mitochondrial fragmentation in tubular cells during acute kidney injury. *Science Signaling*.

[93] Wang J., Toan S., Zhou H. (2020). New insights into the role of mitochondria in cardiac microvascular ischemia/reperfusion injury. *Angiogenesis*.

[94] Pfanner N., Warscheid B., Wiedemann N. (2019). Mitochondrial proteins: from biogenesis to functional networks. *Nature Reviews Molecular Cell Biology*.

[95] Wang J., Chen P., Cao Q., Wang W., Chang X. (2022). Traditional Chinese medicine ginseng Dingzhi decoction ameliorates myocardial fibrosis and high glucose-induced cardiomyocyte injury by regulating intestinal flora and mitochondrial dysfunction. *Oxidative Medicine and Cellular Longevity*.

[96] Chang X., Zhang T., Zhang W., Zhao Z., Sun J. (2020). Natural drugs as a treatment strategy for cardiovascular disease through the regulation of oxidative stress. *Oxidative Medicine and Cellular Longevity*.

[97] Dombi E., Mortiboys H., Poulton J. (2018). Modulating mitophagy in mitochondrial disease. *Current Medicinal Chemistry*.

[98] Bravo-San Pedro J. M., Kroemer G., Galluzzi L. (2017). Autophagy and mitophagy in cardiovascular disease. *Circulation Research*.

[99] Lin Y. F., Haynes C. M. (2016). Metabolism and the UPR^mt^. *Molecular Cell*.

[100] Li Y., Xue Y., Xu X. (2019). A mitochondrial FUNDC1/HSC70 interaction organizes the proteostatic stress response at the risk of cell morbidity. *The EMBO Journal*.

[101] Burman J. L., Pickles S., Wang C. (2017). Mitochondrial fission facilitates the selective mitophagy of protein aggregates. *The Journal of Cell Biology*.

[102] Jin S. M., Youle R. J. (2013). The accumulation of misfolded proteins in the mitochondrial matrix is sensed by PINK1 to induce PARK2/Parkin-mediated mitophagy of polarized mitochondria. *Autophagy*.

[103] van der Giezen M., Tovar J. (2005). Degenerate mitochondria. *EMBO Reports*.

[104] Jin Q., Li R., Hu N. (2018). DUSP1 alleviates cardiac ischemia/reperfusion injury by suppressing the Mff- required mitochondrial fission and Bnip3-related mitophagy via the JNK pathways. *Redox Biology*.

[105] Meyer J. N., Leuthner T. C., Luz A. L. (2017). Mitochondrial fusion, fission, and mitochondrial toxicity. *Toxicology*.

[106] Chan D. C. (2020). Mitochondrial dynamics and its involvement in disease. *Annual Review of Pathology*.

[107] Giacomello M., Pyakurel A., Glytsou C., Scorrano L. (2020). The cell biology of mitochondrial membrane dynamics. *Nature Reviews Molecular Cell Biology*.

[108] Sumneang N., Siri-Angkul N., Kumfu S., Chattipakorn S. C., Chattipakorn N. (2020). The effects of iron overload on mitochondrial function, mitochondrial dynamics, and ferroptosis in cardiomyocytes. *Archives of Biochemistry and Biophysics*.

[109] Sun D., Wang J., Toan S. (2022). Molecular mechanisms of coronary microvascular endothelial dysfunction in diabetes mellitus: focus on mitochondrial quality surveillance. *Angiogenesis*.

[110] Forrester S. J., Preston K. J., Cooper H. A. (2020). Mitochondrial fission mediates Endothelial Inflammation. *Hypertension*.

[111] Xie L. L., Shi F., Tan Z., Li Y., Bode A. M., Cao Y. (2018). Mitochondrial network structure homeostasis and cell death. *Cancer Science*.

[112] Knott A. B., Perkins G., Schwarzenbacher R., Bossy-Wetzel E. (2008). Mitochondrial fragmentation in neurodegeneration. *Nature Reviews Neuroscience*.

[113] Ong S. B., Hausenloy D. J. (2010). Mitochondrial morphology and cardiovascular disease. *Cardiovascular Research*.

[114] Haroon S., Vermulst M. (2016). Linking mitochondrial dynamics to mitochondrial protein quality control. *Current Opinion in Genetics & Development*.

[115] Fu Y., Tigano M., Sfeir A. (2020). Safeguarding mitochondrial genomes in higher eukaryotes. *Nature Structural & Molecular Biology*.

[116] Song J., Herrmann J. M., Becker T. (2021). Quality control of the mitochondrial proteome. *Nature Reviews Molecular Cell Biology*.

[117] Bragoszewski P., Turek M., Chacinska A. (2017). Control of mitochondrial biogenesis and function by the ubiquitin-proteasome system. *Open Biology*.

[118] Rovira-Llopis S., Bañuls C., Diaz-Morales N., Hernandez-Mijares A., Rocha M., Victor V. M. (2017). Mitochondrial dynamics in type 2 diabetes: pathophysiological implications. *Redox Biology*.

[119] van der Bliek A. M., Shen Q., Kawajiri S. (2013). Mechanisms of mitochondrial fission and fusion. *Cold Spring Harbor Perspectives in Biology*.

[120] Chandhok G., Lazarou M., Neumann B. (2018). Structure, function, and regulation of mitofusin-2 in health and disease. *Biological Reviews of the Cambridge Philosophical Society*.

[121] Jin J. Y., Wei X. X., Zhi X. L., Wang X. H., Meng D. (2021). Drp1-dependent mitochondrial fission in cardiovascular disease. *Acta Pharmacologica Sinica*.

[122] Anand R., Wai T., Baker M. J. (2014). The i-AAA protease YME1L and OMA1 cleave OPA1 to balance mitochondrial fusion and fission. *The Journal of Cell Biology*.

[123] Liu K., Ologbenla A., Houry W. A. (2014). Dynamics of the ClpP serine protease: a model for self-compartmentalized proteases. *Critical Reviews in Biochemistry and Molecular Biology*.

[124] Deepa S. S., Bhaskaran S., Ranjit R. (2016). Down-regulation of the mitochondrial matrix peptidase ClpP in muscle cells causes mitochondrial dysfunction and decreases cell proliferation. *Free Radical Biology & Medicine*.

[125] Lin M. T., Beal M. F. (2006). Mitochondrial dysfunction and oxidative stress in neurodegenerative diseases. *Nature*.

[126] Gustafsson C. M., Falkenberg M., Larsson N. G. (2016). Maintenance and expression of mammalian mitochondrial DNA. *Annual Review of Biochemistry*.

[127] Tseng A. H., Shieh S. S., Wang D. L. (2013). SIRT3 deacetylates FOXO3 to protect mitochondria against oxidative damage. *Free Radical Biology & Medicine*.

[128] Mesbah Moosavi Z. S., Hood D. A. (2017). The unfolded protein response in relation to mitochondrial biogenesis in skeletal muscle cells. *American Journal of Physiology Cell Physiology*.

